# Modified management mode for colorectal cancer during COVID-19 outbreak – a single-center experience

**DOI:** 10.18632/aging.103099

**Published:** 2020-05-05

**Authors:** Dexiang Zhu, Qi Wu, Qi Lin, Ye Wei

**Affiliations:** 1 Department of General Surgery, Zhongshan Hospital, Fudan University, Shanghai, China

**Keywords:** COVID-19, colorectal cancer, management model

## Abstract

During the epidemic of COVID-19, the management model of colorectal cancer has to be changed at our center due to relatively limited medical resources. Outpatient visits are reduced under well protected after appointment, and rigorous investigation of epidemiological history and clinical symptoms are needed. We prefer a simple and convenient treatment regimen, which may also be postponed appropriately. Minimally invasive CRC surgery combined with a perioperative program of enhanced recovery after surgery should be recommended. We also focus on mental health treatments and healthy lifestyle education. In addition, routine follow-up can be moderately delayed. In total, adequate doctor-patient communication is also recommended throughout the treatment.

## INTRODUCTION

From the end of 2019, a war without gunpowder has begun in China. Novel coronavirus pneumonia (COVID-19) from Wuhan city has now spread to the whole country and even the world [1]. The major routes of the coronavirus infection are the respiratory droplets, close contact transmission, and also when exposed to high concentrations of aerosol in a relatively closed environment for a long time [2]. The incubation period of COVID-19 is up to 24 days. The most common symptoms were fever and cough, and some severe cases can quickly progress to acute respiratory distress syndrome [3]. The Chinese government has initiated a first-level response to major public health emergencies, mobilized the whole country to fight against the epidemic, made comprehensive deployments, and implemented the strongest and strictest prevention and control measures. By the end of March, the epidemic situation has been under control across China. However, the pandemic of COVID-19 is global now.

Colorectal cancer (CRC) is the malignancy with the fourth highest prevalence among females and fifth among males in China [4]. CRC patients were generally in poor immunity and physical fitness, which are susceptible to COVID-19. A prospective cohort study has found that cancer patients were at higher risk of COVID-19 infection and had a worse prognosis than those without tumors [5].

During this particular period, most hospitals have suspended or postponed outpatient and elective surgery. Therefore, how to deal with CRC patients is challenging and essential. As follows, we introduce our single center's experience in the management of CRC patients during COVID-19 outbreak and present a series of issues of our clinical work (Table 1).

**Table 1:** Changes in clinical practice during COVID-19 outbreak.

**Terms**		**Details**
Outpatient	Appointment	Online
	Escort	One at most
	Protection	A medical surgical mask or a general medical mask at least
	Screening	Check body temperature; Check for signs and symptoms; Epidemiological investigation
	Treatment	Reduce infusion time; Long prescription policy
Non-surgical treatment	Adjuvant chemotherapy	Prefer simple and convenient regimen in principle; Continue the original regimen in principle
	Neoadjuvant therapy	Prefer neoadjuvant chemotherapy alone; Expand neoadjuvant therapy indications for low to moderate locally advanced rectal cancer
	mCRC patient	Develop, improve or change the regimen by online MDT clinics or Wechat group
	Traditional Chinese Medicine	Follow the physicians' advice
Surgery	Before surgery	A comprehensive examination to exclude COVID-19
	Surgery procedures	Prefer minimally invasive surgery plus ERAS program; Not recommend colorectal and liver resection simultaneously; Postpone surgery where condition permits
Daily life		Mental health; Healthy lifestyle
Follow up		Postpone review time appropriately; The principle of proximity hospital; Online follow-up

## Outpatient

If CRC patients have obvious symptoms of bleeding, perforation, obstruction, or extreme discomfort, we recommend them to go to the emergency as soon as possible. Regular outpatient visits can be postponed appropriately. With the joint efforts of the whole country, the epidemic situation has changed positively, and outpatient in various places has gradually restored.

### Appointment

Not as the previous, all outpatient clinics request appointments now. CRC patients should make an appointment in advance, and only one family member is allowed to accompany to prevent cross-infection.

### Protection

The medical workers, patients and families need to strengthen their own protection, such as wearing surgical masks, goggles, and so on. We also pay attention to the disinfection of outpatient equipment and the environment.

### Epidemiological history

At the clinic, a rigorous investigation of epidemiological history and clinical symptoms is needed. Patients with a history of living or traveling in the affected area, close contacts with a confirmed or probable case, or having fever and respiratory symptoms, should be checked during pre-diagnosis. And then, if suspected COVID-19 manifestations, the patient should be sent to the fever clinic. If a suspected or confirmed case is diagnosed, the patient shall be immediately quarantined and reported.

### Outpatient treatment

At the outpatient, we choose simple and convenient regimen, and also establish a long prescription policy to facilitate patients to receive drugs for 2 to 3 months at a time, to reduce the times of visiting.

## Non-surgical treatment

The Chinese Society of Clinical Oncology guidelines recommend that adjuvant chemotherapy should be started as soon as possible after recovery, generally about 3 weeks after CRC operation, and no later than 2 months [6]. A meta-analysis of 15 410 CRC patients showed that the start of postoperative adjuvant chemotherapy was delayed every 4 weeks, patients' overall survival time and disease-free survival time will be significantly reduced [7]. Therefore, we recommend postponing adjuvant chemotherapy appropriately at the local hospital as the first choice. Moreover, we prefer the three-week CapeOX regimen to biweekly FOLFOX regimen, so that we can minimize the chance of cross-infection. Oral capecitabine monotherapy also could be used as much as possible. In addition, we recommend that the patients can contact the physicians to reduce the treatment intensity and switch to oral therapy.

During the epidemic, many hospitals suspended radiotherapy. The Chinese FOWARC Trial showed that no significant difference in outcomes was found between mFOLFOX6 without radiotherapy and fluorouracil with radiotherapy for locally advanced rectal cancer [8]. Therefore, neoadjuvant chemotherapy alone with the mFOLFOX6 regimen is also an option.

For patients with metastatic CRC, Multi-Disciplinary Treatment (MDT) is the best choice. If the condition is stable, the original chemotherapy regimen can be maintained for another 1-2 cycles until the MDT outpatient restore. If there is obvious progression, we recommend online MDT clinics or communicating with physicians via WeChat or telephone to change the regimen.

Undoubtedly, traditional Chinese medicine has certain effects on improving the physical condition of CRC patients, which can reduce the side effects of chemotherapy and improve the quality of life [9]. Considering the patients' resistance during chemotherapy is relatively low, we also recommend regular thymosin to improve immunity as prescribed [10].

## Surgery

CRC surgeons should control the routine operation to reduce the patient's exposure time in the hospital [11]. For CRC patients with mass bleeding, perforation, or obstruction, emergency surgery should be considered, and COVID-19 infection needs to be ruled out before.

Endoscopic surgery is recommended for early-stage CRC when it is completely removed clearly with good histological features, and no additional surgical treatment is required. Whether surgery delay affects survival remains controversial for advanced CRC. We prefer to expand neoadjuvant therapy indications for low to moderate locally advanced rectal cancer. And we try to conduct surgery for advanced colon cancer as early as possible. A detailed investigation and a comprehensive examination (chest CT or viral nucleic acid test) should be performed to exclude COVID-19 before elective surgery. In addition, we also do not recommend to perform colorectal and liver resections simultaneously at the current situation, so as to avoid a prolonged hospital stay and increased risk of infection.

At the surgical ward, we prefer minimally invasive surgery plus a perioperative program of enhanced recovery after surgery (ERAS) as the best treatment strategy, which could accelerate patient recovery and shorten hospital stay [12].

The hospital should strictly implement the National Health Commission's requirements for infection control in medical institutions [13]. Ordinary patients who underwent CRC resection can be transferred to the general ward after surgery. It is necessary to reduce the movement of accompanying staff and personnel. Patients with postoperative fever should be carefully identified and isolated according to the suspected COVID-19 criteria [2]. And then suspected or confirmed patients should be transferred to a designated negative pressure isolation monitoring room for single room isolation.

## Daily life

CRC and COVID-19 are double blows to patients. Many patients have mental health problems of anxiety and depression, so we should give positive psychological support during the epidemic. We inform that the prognosis of CRC is not so bad, and even with recurrence, a considerable part of the patients will be cured when metastases are detected and resected early [14]. Stoma patients are also encouraged to communicate with families and friends, learn to self-regulate bad moods, and actively integrate into society. At the same time, we also inform the patients that they will not be infected if actively protect, and the epidemic situation is getting better now, which will return to normal soon. Even if the treatment is appropriately delayed, it will not affect the treatment effect. At present, several public institutions and domestic hospitals have launched psychological hotline services, and we recommend patients could contact when needed.

A healthy lifestyle is especially important for CRC patients. Studies have shown that smokers have a significantly increased risk of developing and dying from CRC compared with never-smokers, and heavy drinking also increases the risk of developing CRC [15]. We recommend CRC patients a healthy and balanced lifestyle diet, avoiding high fat and low fiber diet, reducing the intake of red meat and processed meat. The American Gastroenterological Association has recommended calcium supplements for the primary or secondary prevention of colon cancer, so we also recommend appropriate intake of calcium-rich food such as dairy products [16]. CRC patients also need to appropriately increase the intake of cellulose and decrease irritating and too much oily food. Stoma patients can properly consume dairy products and vegetables to reduce the odor at the stoma. In addition, we also suggest that during the epidemic, patients can arrange indoor physical exercise under the guidance of the physicians, avoiding prolonged bed rest, which can promote the recovery of intestinal function and prevent deep vein thrombosis.

Recently, many health organizers have opened public-interest online lectures and free mobile applications for different patient groups, to provide disease education and answer questions online. Furthermore, several university hospitals have also opened up various online clinics, including online fever clinics, psychological clinics, specialist clinics and online MDT clinics. In addition, we also have established several follow-up WeChat groups for CRC patients. When CRC patients have questions, they can get medical advice quickly from multiple experts at home.

## Follow up

For patients who need to be reviewed after CRC surgery during the epidemic, we recommend that the review time can be appropriately postponed. We encourage patients to complete routine review projects at the nearest medical institution. After they obtain the review results, it is recommended to adopt an online network method for consultation.

In summary, our current clinical work model has to be changed due to COVID-19 outbreak, which includes the above outpatient, inpatient, psychological treatment and health education.
